# Treatment of simple bone cysts using endoscopic curettage: a case series analysis

**DOI:** 10.1186/s13018-018-0869-z

**Published:** 2018-07-05

**Authors:** Hisaki Aiba, Masaaki Kobayashi, Yuko Waguri-Nagaya, Hideyuki Goto, Jun Mizutani, Satoshi Yamada, Hideki Okamoto, Masahiro Nozaki, Hiroto Mitsui, Shinji Miwa, Makoto Kobayashi, Kojiro Endo, Shiro Saito, Taeko Goto, Takanobu Otsuka

**Affiliations:** 1 Department of Orthopedic Surgery, Nagoya City University Graduate School of Medical Sciences, 1, Kawasumi, Mizuho-cho, Mizuho-ku, Nagoya, 467-8601 Japan; 2 Department of Joint Surgery for Rheumatic Diseases, Nagoya City University Graduate School of Medical Sciences, 1, Kawasumi, Mizuho-cho, Mizuho-ku, Nagoya, 467-8601 Japan; 3 Department of Rehabilitation Medicine, Nagoya City University Graduate School of Medical Sciences, 1, Kawasumi, Mizuho-cho, Mizuho-ku, Nagoya, 467-8601 Japan; 4 Department of Radiology, Nagoya City University Graduate School of Medical Sciences, 1, Kawasumi, Mizuho-cho, Mizuho-ku, Nagoya, 467-8601 Japan; 5 Department of Orthopedic Surgery, Ogaki Municipal Hospital, 4-86 Minaminokawa-cho, Ogaki, 503-8502 Japan

**Keywords:** Endoscopy, Endoscopic curettage, Simple bone cysts, Recurrence, Minimally invasive procedure

## Abstract

**Background:**

Endoscopic curettage is considered applicable for the treatment of simple bone cysts with the expectation that it might be less invasive than open curettage. In this study, we investigated the efficacy of endoscopic curettage for the treatment of simple bone cysts. The goal was to investigate the incidence of cyst recurrence and bone healing after endoscopic curettage. Moreover, complications and functionality at the final follow-up were evaluated.

**Methods:**

From 2003 to 2014, 37 patients with simple bone cysts underwent endoscopic curettage. Twenty-four were male and 13 were female, with a mean age of 14.7 years. Endoscopic curettage was performed with the support of an arthroscope via 7–8 mm holes penetrated by cannulated drills with a small incision. The cysts underwent curettage using angled curettes, rongeurs, and an electrical shaver until the normal bone was observed in the medullary cavity. To investigate the bone healing after endoscopic curettage, we evaluated the consolidation of the cyst at the final evaluation (Modified Neer Classification) and the time to solid union after operation, which was defined as the sufficient thickness of the cortical bone to prevent fracture and allow physical activities.

**Results:**

Recurrence occurred in seven patients (18.9%). A log-rank analysis revealed that contact with the physis was associated with recurrence (*p* = 0.006). Among 31 patients (83.7%), the consolidation of cyst was considered healed at the final X-ray follow-up period, and in these patients, the mean time taken for solid union of cortical bone thinning was 4.0 months (standard deviation, 2.4). With regard to major complications of endoscopic curettage, a transient radial nerve palsy and two postoperative fractures occurred. The former problem was managed conservatively and the latter problems by transient internal fixation; these problems were managed without any further complications. All patients had a good postoperative function.

**Conclusions:**

Endoscopic curettage might be a useful alternative as it is a minimally invasive procedure for the treatment of simple bone cysts. Considering the relatively smaller size of this study, further investigation should be necessary for deducing the reliable conclusion.

## Background

Simple bone cysts (SBCs) are benign bone tumors that arise mainly at the proximal humerus, femur, or calcaneus [1]. The cystic cavity is filled with serous or serosanguineous fluid and lined by thin fibrovascular connective tissue membrane. The etiology of this lesion has been enigmatic; however, obstruction from venous outflow might be one of the reasons that a cystic cavity fills with fluid. Moreover, the disturbance in growth at the epiphysial plate was considered the cause of cystic cavity [2]. Usually, these cysts weaken the cortex, predisposing the bone to pathologic fracture [3]. SBCs are most commonly found in adolescent from birth to 20 years of age [4].

Although there still have not been a study with a high level of evidence and comparison among each treatment is difficult, many methods of treatment have been introduced, including observation [5], steroid injections [6], curettage with or without bone grafting [7], or insertion of a cannulated pin/screw [8, 9]. Each management method has pros and cons. The observation method requires a relatively longer time for consolidation of the cystic cavity and prolonged restriction of activity to prevent pathologic fractures [10]. Steroid injection is a non-operative treatment and was proven to be superior to bone marrow injection in the randomized controlled trial, but it sometimes required multiple procedures because of low healing rate [11]. The open curettage with or without bone grafting is still the cornerstone for treatment and superior to steroid injection in terms of healing rate as demonstrated in the retrospective comparative study performed by Sung et al. [3]. However, this technique is too aggressive with occasional postoperative complications and recurrence after surgery sometimes occurs. Decompression of cystic cavity by making holes using multiple drilling [12] or insertion of a cannulated pin or screw conforms the etiology of the SBC. However, there are still lacking evidence and soft tissue damage to access the bone surface that cannot be ignored. Moreover, inserted pins can sometimes be dislodged from the cannulated bone, which leads to irritation of tissues surrounding the pin [8, 9].

However, eventual healing of SBC is assured in many cases; some patients require multiple attempts to deal with a recurrence; thus, minimal invasiveness per treatment is especially desired. Here, we introduce endoscopic curettage (ESC) for SBC with the expectation that this procedure will have lower invasiveness and relatively higher success rate compared to other traditional procedures and elaborate on our surgical and clinical outcomes with this treatment approach.

## Methods

### Patient characteristics

From 2003 to 2014, we extracted the data of 40 patients histologically diagnosed as having SBCs. The analyses of their specimens were conducted at the Division of Pathology of Nagoya City University. Among these patients, three patients aged over 30 years were excluded from the analysis because the etiology of older patients might be different [2, 13]. Finally, a total of 24 male and 13 female patients, with a mean age of 14.7 (standard deviation [SD], 6.3) years, were included in the study. The tumors were located in 29 tubular bones (18 humeri, 8 femurs, 2 radii, and 1 tibia), 2 flat bones (pelvises), and 6 short bones (calcanei). All patients were monitored for at least 6 months after solid union and the consolidation of bone (Modified Neer Classification A–B [described in postoperative evaluation section]). In cases in which the cyst did not consolidate over time (Modified Neer Classification C–D), follow-up periods were extended to at least 3 years after the operation. The mean follow-up period was 33.8 (SD 26.1) months.

### Surgical procedure

All surgical procedures were conducted or supervised by MK. The localization of the tumor was identified with an image intensifier, and marks were drawn on the skin along the edge of the tumor. The number of portals needed depended on the size of the tumor and location.

The surgical procedure was performed with the aid of an inflatable tourniquet. In cases in which the tourniquet was not applicable (e.g., tumors located in the proximal humerus, pelvis, or proximal femur), epinephrine (equivalent to 3.3 mg/L) was added into the irrigation fluid to control the blood loss.

**Fig. 1:**
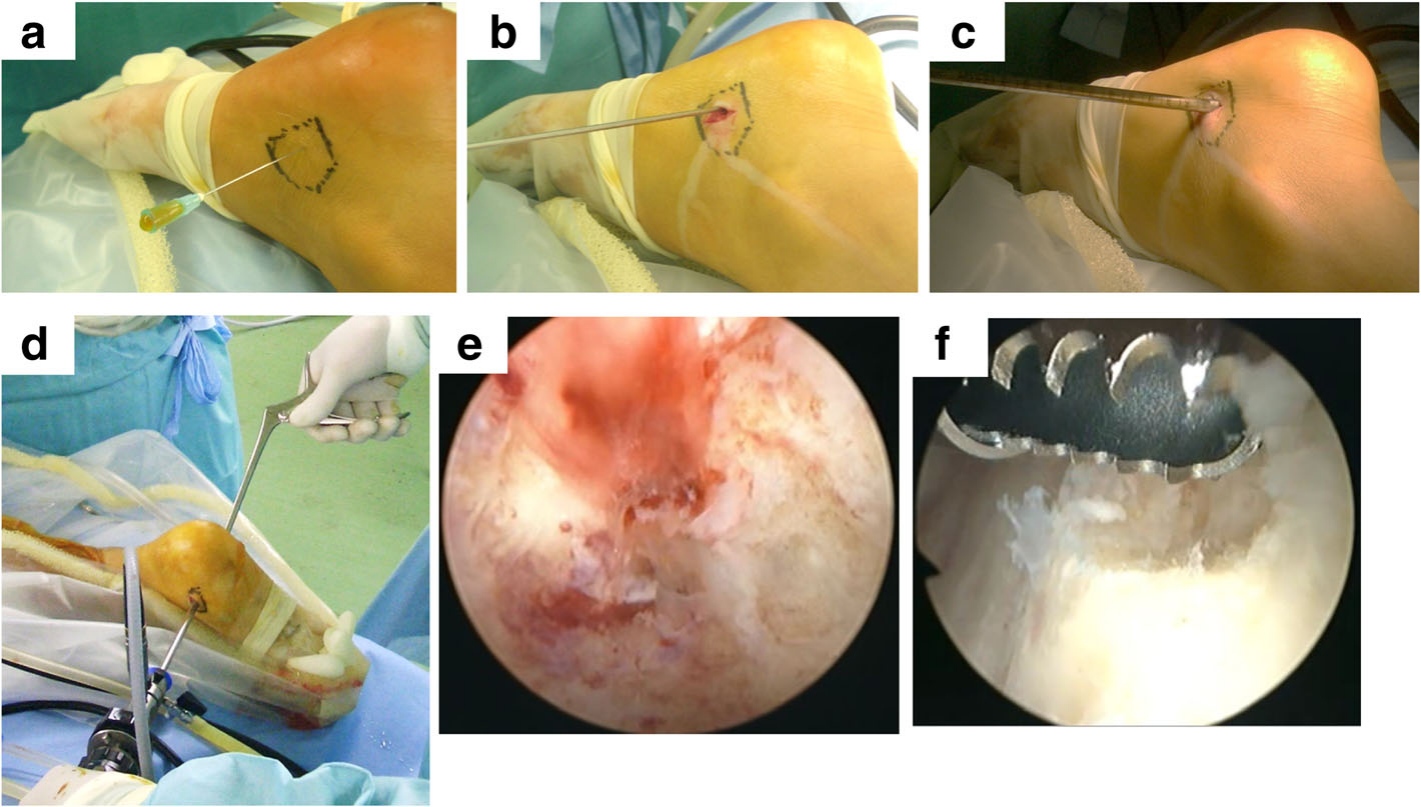
The intraoperative findings were obtained during surgery for simple bone cyst in the right calcaneus. Collection of intracavity fluid (**a**). Penetration of bone with Kirschner wire (**b**). Step-wise cannulation of the small hole (**c**). Surgical maneuver (**d**). Intracavity findings before curettage (**e**). Normal cortical bone after complete curettage (**f**)

**Fig. 2:**
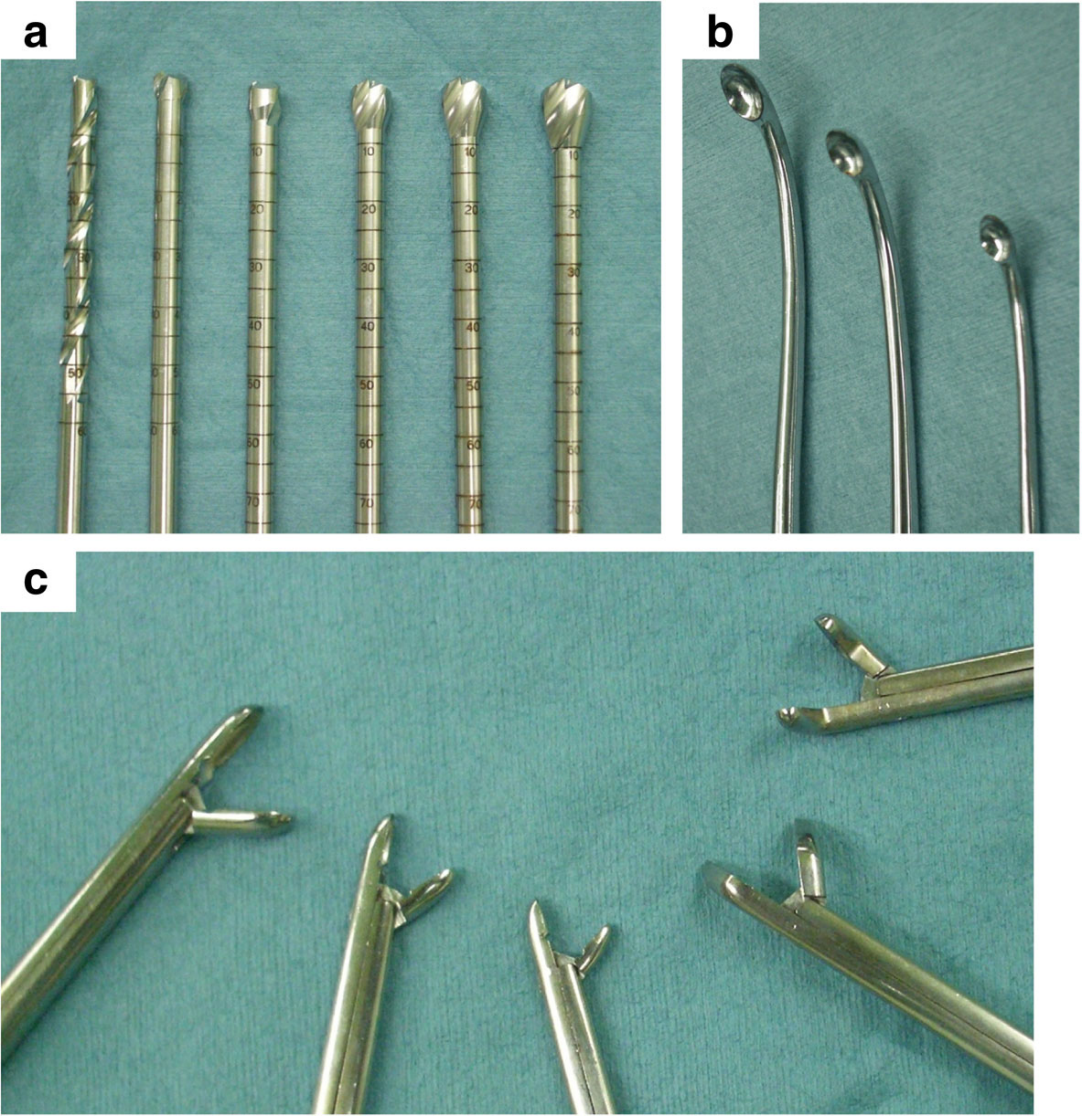
Surgical instruments. Step-up cannulated drills (**a**). Variously angled curettes (**b**). Angled forceps (**c**)

After making an approximately 1-cm incision in the skin, the soft tissue was bluntly dissected until reaching the bone surface. The cortical bone was pierced with a 2.0-mm Kirschner wire, and intraosseous fluid was obtained through the pierced bone and observed for its color and properties (Figs. 1a, b). The small bone hole was enlarged using step-up cannulated drills up to 7 or 8 mm (Figs. 1c and 2a). Subsequently, the second portal was made in the same fashion, and if necessary, a third or fourth one was made. An arthroscope (usually 4 mm in diameter, or 2.7 mm in the case of cysts in a small bone) was inserted into the cavity of the cyst to observe the inside of the cyst.

Under endoscopic visualization, the surgical instruments, including arthroscopic curette (Fig. 2b) or forceps (Fig. 2c), were inserted through the portal at various angles (Fig. 1d). The arthroscope and surgical instruments were exchanged to ensure complete observation of the cavity. The cystic lesion was thoroughly removed until the normal bone was seen in the medullary cavity (Fig. 1e, f). In cases with multiple compartments in the cavity, the septa are resected using shavers. By opening the separated compartments, reduction of internal pressure of each cyst is achieved. The blind area in the vicinity of the endoscope portals was carefully observed.

In the postoperative periods, the patients with lower limb lesions were limited to bearing half of their weight by using a crutch for 1 month, followed by 1 month of two-thirds partial weight bearing with a crutch, and full weight bearing only after 3 months if bone healing was confirmed. On the other hand, the patients with upper limb or non-weight-bearing bone cysts had no limitations of any activities, except for contact sports, until solid union.

### Postoperative evaluation

Complication rate and functionality (assessed in terms of the Musculoskeletal Tumor Society Score) were evaluated after treatment [14]. As for the bone healing after ESC, we evaluated solid union, which was defined by the method of Hou et al. [15]: “the cortical wall thickness was sufficient to prevent further fracture and allowed unrestricted physical activity.” Moreover, the consolidation of the cyst at the final evaluation was assessed according to the Modified Neer Classification [13] (Table 1), by an orthopedic surgeon and radiologist with independent of patients’ information.

**Table 1:** Modified Neer Classification [13]

Classification	Description	Details
A	Healed	Cyst filled with new bone with small radiolucent area (< 1 cm)
B	Healed with a defect	Radiolucent area (< 50% diameter) with enough cortical thickness
C	Persistent cyst	Radiolucent area (≧ 50% diameter) with thin cortical rim
D	Recurrent cyst	Cyst reappears in the obliterated area or increased residual radiolucent area

### Statistical analysis

Kaplan-Meier analysis and the log-rank test were used to determine the association between recurrence and patient variables. Radiographical images (Modified Neer Classification) at the final assessment were evaluated independently and cross-checked by an orthopedic surgeon (HA) and radiologist (TG) who were both blinded to all other patient information. The kappa value was then calculated. For the statistical analysis of the categorical data, the chi-square analysis was used. A *p* value < 0.05 was considered significant. All values were presented either as mean ± standard deviation or median with range, depending on the distribution. All statistical analyses were conducted using SPSS version 24 (IBM, Chicago, IL).

## Results

**Fig. 3:**
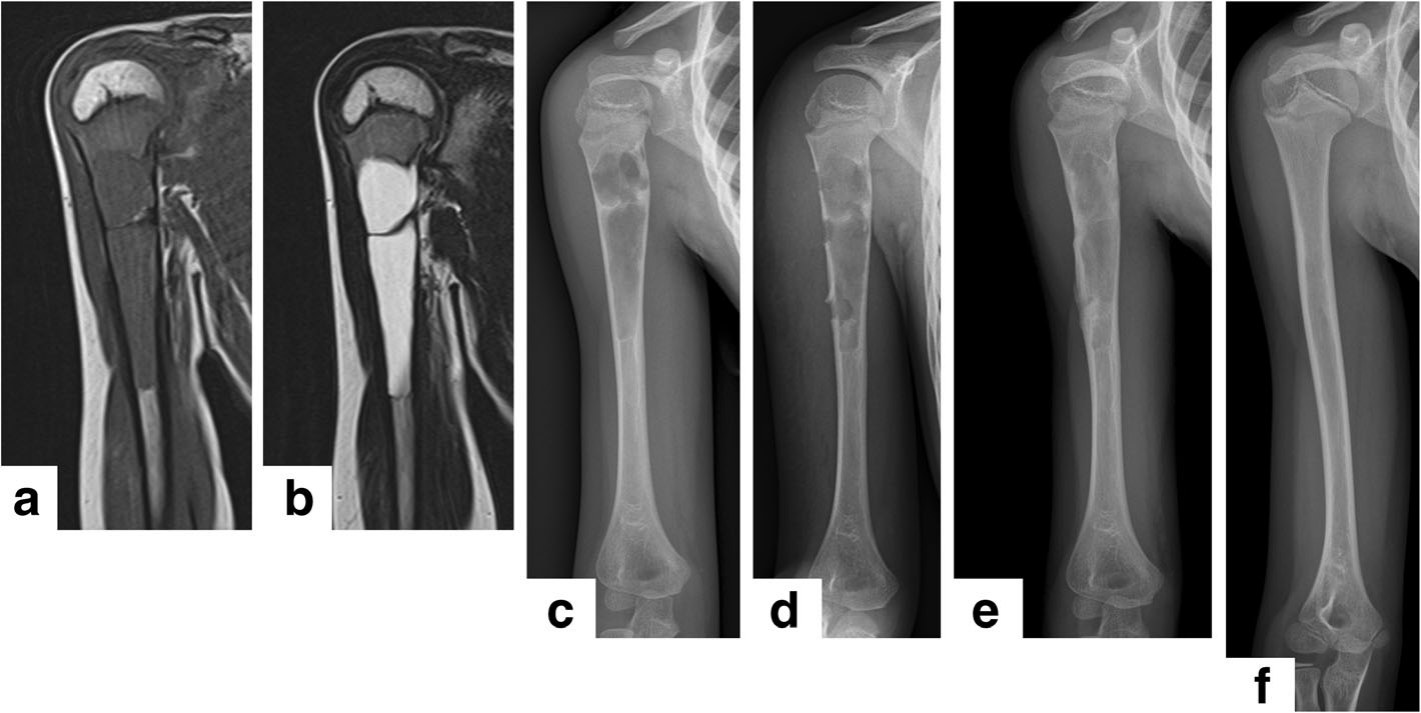
The typical case of simple bone cyst in the right humerus treated with endoscopic curettage. A 6-year-old boy bruised his shoulder (**a** T1-weighted magnetic resonance [MRI]; **b** T2-weighted MRI; **c** X-ray). After 6 months of conservative therapy, the patient underwent endoscopic curettage via three portals (**d** postoperative image). Three months after the procedure, healing was confirmed with a callus around the portals and consolidation in the cavity (**e** solid union). Three years later, the bone was remodeled without any residual tumor or angular deformity (**f** class A)

**Fig. 4:**
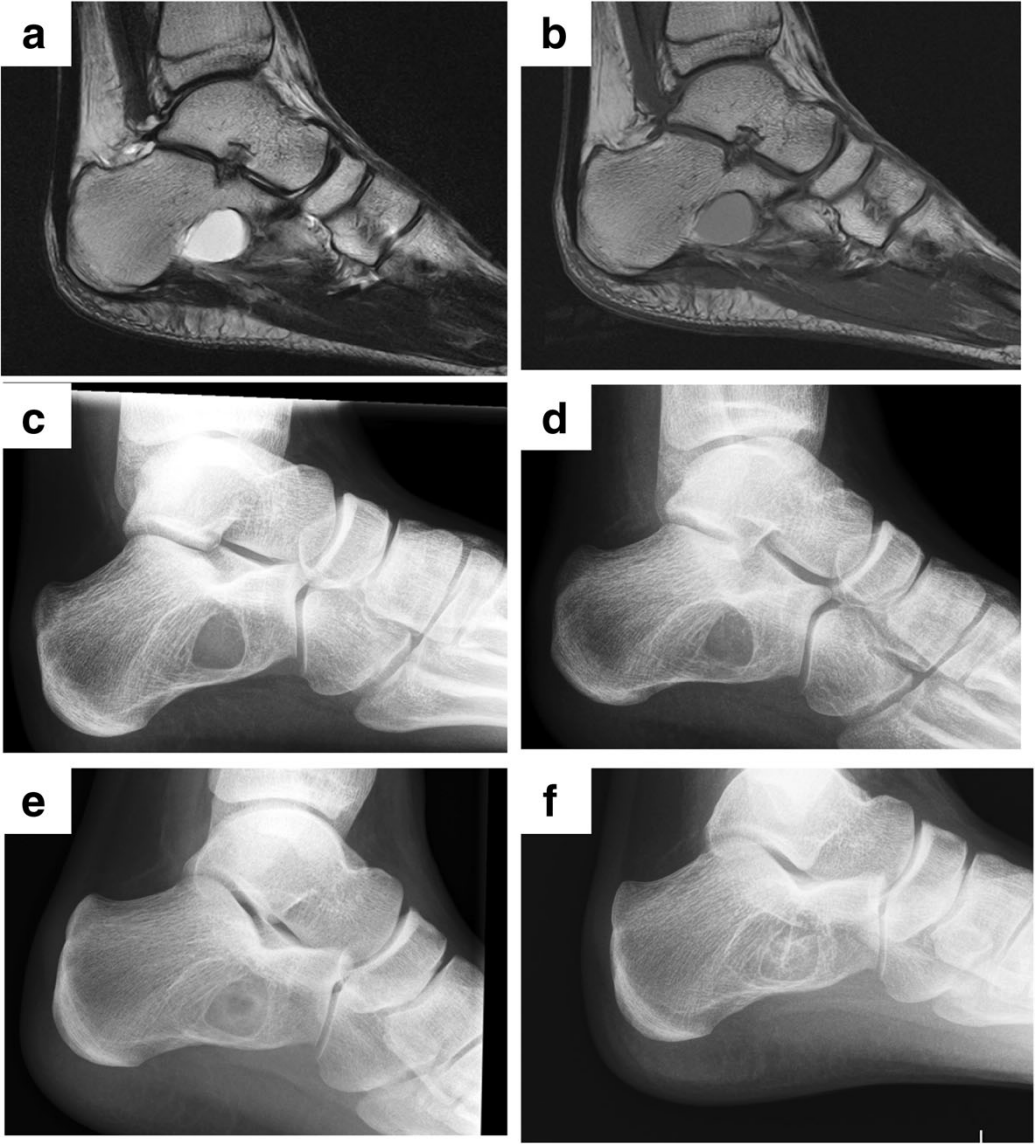
Typical case of simple bone cyst in the calcaneus treated with endoscopic curettage. An 8-year-old boy experienced heel pain without an apparent cause (**a** T1-weighted MRI; **b** T2-weighted MRI; **c** X-ray). Endoscopic curettage C was performed via two portals (**d** postoperative image). After 3 months, healing was confirmed with consolidation of the cyst (**e**), and the cavity was completely filled with new bone 6 years after the operation (**f**, class A)

ESCs were performed from 2.5 portals on average (4 portals in 3 cases; 3 portals in 11 cases; 2 portals in 23 cases). The number of portals depended on the length of the lesion to ensure an appropriate working space for curettage and better viewing area. The median operative time was 88.8 (range, 42.0 to 186.0) min. The median volume of intraoperative bleeding, estimated from the total amount of irrigation fluid, was 21.7 ml (range, almost zero to 205.0 ml). Typical cases of SBC treated with ESC are shown in Figs. 3 and 4.

### Recurrence after ESC

During follow-up, recurrence occurred in seven patients (18.9%), at a median of 17.5 months (range, 7.8 to 28.8 months) after surgery. Among patients with recurrent cysts (*n* = 7), four patients underwent ESC again, and thereafter, the cystic lesions were well managed; two patients requested open curettage and artificial bone graft (OSferion®, Olympus Co., Tokyo, Japan); and one patient without any symptoms was observed. From the log-rank analysis, recurrence was associated with continuity of the cyst to the physis (*p* = 0.006, Table 2).

**Table 2:** Association between variables and recurrence

Variables	Number of cases (*N* = 37)	Recurrence	*p* value; chi-square analysis
Age, year			0.103
< 10	7	3	
≧ 10	30	4	
Sex			0.567
Male	24	4	
Female	13	3	
Location*			0.285
Tubular bone	29	6	
Flat bone	2	1	
Short bone	6	0	
Contact with physis			0.006
Yes	12	4	
No	25	3	
Maximum length of tumor			0.471
≧ 50 mm	14	2	
< 50 mm	23	5	

*Comparison for pooled over strata

### Healing after ESC

A total of 31 patients (83.7%) were categorized as healed (class A or B), and of these patients, the mean time of healing after the operation was 4.0 (SD 2.4) months. The kappa value was substantial (0.64, *p* = 0.002) between the two observers. The residue of the cyst was associated with ages under 10 years (*p* = 0.034, chi-square analysis). Despite there being no significant difference, cyst residue did not occur in patients with the cyst located in the calcaneus (*p* = 0.239; chi-square analysis of calcaneus to other bones).

### Complications and function after ESC

**Fig. 5:**
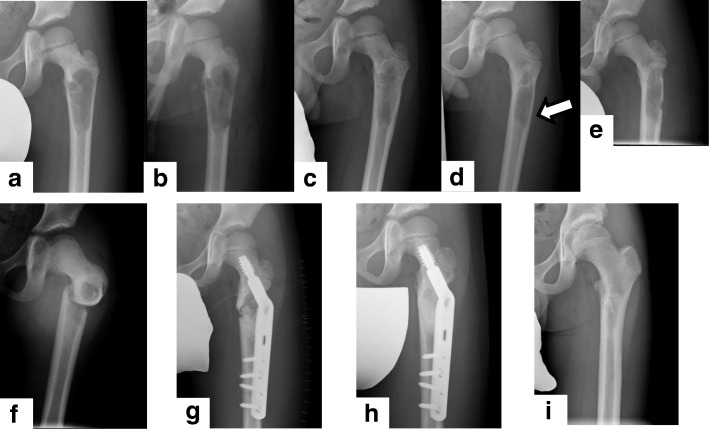
Recurrence and pathologic fracture after second endoscopic curettage. A 5-year-old boy had left coxalgia; from the X-ray image (**a**), simple bone cyst was suspected. The first endoscopic curettage was performed via two portals (**b**). After 2 months, bone healing had begun with cortical enlargement and consolidation of cancellous bone (**c**). However, approximately 1 year after the endoscopic curettage, the cystic lesion (white arrow) had become prominent and recurrence was suspected (**d**). A second endoscopic curettage was performed (**e**). After discharge with a crutch, the patient fell, and a subtrochanteric fracture was identified (**f**). Open reduction and internal fixation with a compression hip screw (Ti-VFx II tube plate®, Zimmer Biomet, Warsaw, USA) and artificial bone grafting (OSferion®, OLYMPUS) were performed (**g**), and after 6 months, bone union was confirmed (**h**) and the implants removed. Three years after the first endoscopic curettage, no recurrence or complications had occurred (**i**)

All patients had an excellent function after ESC. With regard to minor complications in the humerus, two patients retained a slight deformity, without any symptoms, that was related to the dislocation of the pathological fracture before their first visit. Transient radial nerve palsy occurred for a patient with a large cystic lesion across the shaft of the humerus, probably due to iatrogenic blunt compression of the radial nerve during the ESC. Six months later, the palsy had spontaneously recovered without any deficit. Moreover, two postoperative fractures occurred (Fig. 5) and required temporal internal fixation (for 6 and 13 months).

## Discussion

Although the etiology of a unicameral bone cyst has not been fully elucidated, conventional treatments have been to decompress the intraosseous pressure; irrigate the cyst to decrease any bone-destroying enzymes, such as prostaglandin-E2 or gelatinase [16]; remove the cyst membrane; and stimulate the bone healing process [15].

Historically, the percutaneous injection of steroids and aspiration of the fluid in the cavity were first reported by Scaglietti et al. [17]. Although this procedure is not highly invasive, multiple injections are required, and the recurrence rate is high. In 1986, Campanacci et al. [18] reported that 32% of patients with bone cyst had a recurrence or no change after the first treatment. Chang et al. [19] reported that 49% of patients of bone cyst required subsequent injections of steroids, and 44% of patients who had a second injection ended up with failure. In 2007, Wright et al. reported the result of steroid injection therapy by randomized control trial with a comparison to autologous bone marrow injection, revealing the superiority of steroid injection. The healing rate of steroid injection cohort was 42%, and the subsequent fracture rate occurred in 11 cases per 38 patients [11].

Open curettage of the bone cyst with bone grafting, including autograft, allograft, or hydroxyapatite bone substitute, increases the healing rate after surgery. This treatment is simple and easy to learn, but some authors do not recommend it as the initial treatment [3] due to concerns of donor-site infection, iatrogenic fracture, or growth plate injury. Neer et al. reported the outcomes of open curettage and bone grafting with comparison to conservative therapy (immobilization after pathological fracture) for SBC and reported its superiority in terms of healing rate (77 vs 4%, open curettage vs conservative therapy, respectively) and subsequent fracture rate (2.4 vs 80%, respectively) [13]. In 1986, Campanacci et al. reported the outcomes about the bone curettage with bone grafting for a large number of cases (*n* = 178), and the healing rate was 68% [18]. Recently, Sung et al. reported in their retrospective comparative study, the outcomes including treatment failure (defined clinically as a subsequent pathologic fracture or need for retreatment to prevent pathologic fracture) and complications. After curettage and bone grafting, 64% of the patients experienced treatment failure and 21% complained about pain [3].

Given that the optimal treatment for SBC has remained questionable, a meta-analysis integrated 62 articles with a total of 3217 patients with SBC [1]. According to the review, the failure rate—defined as recurrence or persistence of cyst—was 23.9% (overall), with a rate of 61.1% for conservative treatment, 23.2% for curettage with autograft, and 28.5% for methylprednisolone acetate injection. This indicated that the recurrence of 18.9% after ESC in this study was at least not lower compared to that in other studies. However, there may be many factors that influence the results; thus, the comparison of the results between this work and previous studies is difficult. In other words, selection of treatment must be based on various factors, including invasiveness, reproducibility, recurrence rate, and comorbidities.

ESC for benign bone tumors, including SBCs, enchondromas, and aneurysmal bone cysts, has been performed in our institution since the early 1990s [20–22]. We have reported favorable outcomes for patients with enchondromas treated with ESC between 1992 and 2016, with a recurrence rate of 3.3% among 120 patients and a mean time for bone healing of 2.9 months without postoperative contracture [20]. Until now, the report about ESC for SBC was limited, except for the lesion arising from the calcaneus. A pilot study comparing open versus endoscopic curettage and bone grafting was conducted by Yildirim et al. in 2011 [23]. This study included 26 patients, who were equally assigned into two groups. They reported similar healing rates (92.3 and 100% for open curettage and ESC with bone grafting, respectively), and direct visualization and less soft tissue damage were the advantages of this technique. Nishimura et al. also reported a comparative study of the result of ESC with calcium phosphate cement for the calcaneus lesion, which revealed that there was no recurrence and time to sports activity was rapid in the ESC group [24].

Despite the several case reports about ESC for SBC in the tubular bone [25–27], there had never been reports of ESC in a relatively large case series. We have determined that arthroscopy provides an accurate assessment of tumor resection via direct examination of the bone marrow cavity for the complete removal of the cyst and is less invasive compared to the other methods. Likewise, Choi et al. [27] reported the outcomes of endoscopic curettage for various benign bone tumors. They stated that the small number of incisions (two incisions in most cases) had many advantages, such as less bleeding and less damage to the adjacent soft tissue and bone. Furthermore, blind drilling or excessive curettage, which could lead to intraoperative fracture or brittleness of the treated bone, can be avoided [28]. However, we should note that the learning curve or skill of the surgeon might affect the outcomes of ESC; in other words, surgeons must have an experience in both arthroscopic surgery and treatment of bone tumor. Thus, standardization of this treatment for every institution might be difficult, but we believe that ESC can be applied for many treatments, apart from SBC.

Regarding recurrence after surgery, we reported that the risk factor for the recurrence was the attachment to physis. This is probably related to the activity of SBC; the cyst separated from the physis is considered latent [29]. In addition, other reports documented that the recurrence is more likely in patients younger than 10 years old [13, 29] and larger cysts are at a higher risk of recurrence [30, 31]. Moreover, multilocular cysts are more likely to recur [18, 32] as curettage may leave some areas behind. In our series, we performed complete resections of the septa of the multilocular cysts, which can prevent the recurrence of these lesions.

In this study, some major complications of surgery occurred in four patients. One patient had transient nerve palsy, probably caused by iatrogenic blunt compression of the nerve during the procedure around the radial groove. To minimize the complications with ESC, thorough planning before the operation to determine the location of the access ports is needed to avoid neurovascular structures. Additionally, two patients had postoperative fractures early in the study period. Because the strength of the bone might be transiently weakened after curettage of the cavity, careful observation and rigid prohibition of weight bearing are necessary until considerable bone formation, especially in cases in which the cyst was located in the lower extremities. Considering the previous reviews about postoperative fractures with incidence rates of 0–20% for open curettage and 0–30% for steroid injection [33], the fracture rate is within the range of the previous study. However, the prediction of postoperative fracture is not easy because many factors influenced bone strength. Ahn and Park noted from the analysis of SBC and aneurysmal bone cysts that destruction of > 85% of the length of the cortex in the transverse plane on both anteroposterior and lateral views was a risk factor for pathological fracture [34]. Furthermore, Nakamura et al. reported that cyst wall thickness, with a cutoff value of 0.5 mm, was an important risk factor for pathological fractures [35]. However, the specificity of each threshold was not very high. In addition to the length or cyst wall thickness, we deemed that a high lesion/cortex ratio (the ratio between the maximum width of the lesion to the bone [36] and the cyst located in the lower extremity or trochanteric area should be carefully considered if ESC is planned.

This study had several limitations. First, to evaluate the outcomes of this treatment more precisely, comparison with other treatments should have been performed. Second, the sample size was limited; thus, the power of this study is not strong. Finally, validation of the duration of follow-up was another concern in this study. Teoh also reported that the time to recurrence was variable, ranging from 2 to 27 months after surgery [29]. In this study, all patients were monitored for at least 6 months after bone consolidation (Modified Neer Classification A–B). This is because, once the cystic cavity was fully consolidated, recurrence was unusual. However, three of our patients showed late recurrences (22–29 months after the operation). Clinically, these cases of recurrence involved persistent cysts (Modified Neer Classification C). Therefore, if bone healing was delayed, an extension of the follow-up period would be necessary. However, because of the slow-growing nature of the latent residual cysts, the exact determination of recurrence using plain radiography was sometimes difficult; therefore, clinicians sometimes had difficulty in determining when follow-up could be discontinued.

## Conclusions

We have reported a relatively large case series of SBC treated with ESC. ESC might be a useful alternative as it is a minimally invasive procedure for the treatment of simple bone cysts. Considering the relatively smaller size of this study, further investigation should be necessary for deducing the reliable conclusion.

## Abbreviations

ESC: Endoscopic curettage
SBC: Simple bone cyst
SD: Standard deviation
